# Clinical Lectures on Diseases of the Eye

**Published:** 1866-11

**Authors:** E. L. Holmes

**Affiliations:** Lecturer on Diseases of the Eye and Ear in Rush Medical College


*Clinical Lectures on Diseases of the Eye.* By E. L. Holmes, M. D., Lecturer on Diseases of the Eye and Ear in Rush Medical College.

### STRABISMUS.

Gentlemen,—I scarcely need dwell upon the description of the operation for strabismus which you have just seen performed. The different steps in the operation have been several times explained during the course; and yet, as this is probably the last opportunity we shall have of alluding to the subject this winter, I desire to call your attention to several important points. Although there are various modifications in the operation, which you can find described in different works on ophthalmology, I advise you to confine yourselves to the ordinary method, till you have acquired actual experience in operating.

First, therefore, after separating the lids by the aid of the spring speculum or levateurs, and, when necessary, rotating the eye to a convenient position by means of a delicate hook, you seize a horizontal fold of the conjunctiva, snip it with the scissors in such a way that there will be a vertical incision about a third of an inch in length through the mucous membrane, and two or three lines from the cornea; second, after dissecting carefully the cellular tissue to the sclerotic, you pass the blunt hook under the tendon of the muscle and divide it close to the sclerotic. Instead of the blunt hook, passed under the muscle, there is good authority for using a small pair of toothed forceps to seize and slightly elevate the tendon previous to severing it. In this way, there is less pain and less extensive separation of the conjunctiva from the sclerotic than when the blunt hook is used. The dressing necessary after this operation, is simply wet compresses laid over the lids for a few hours. It is well to exclude the patient from exposure to changes of temperature and from use of the eyes for some days. The extravasation of blood into the cellular tissue of the conjunctiva often occurs, but need cause no apprehension. Occasionally, cicatrization takes place slowly, in consequence of the formation of a button of granulations in the wound. This can be snipped off with a pair of scissors.

As regards the steps of the operation, there are perhaps but two points to which there is particular necessity of caution.

The first is, you should not attempt to introduce the blunt hook till the mucous membrane has been fully divided down to the sclerotic. The second is, in dividing the tendon of the muscle, that you should avoid injuring the sclerotic by the imprudent use of too sharp-pointed scissors.

Although the operation is comparatively simple, it is important that great delicacy in the use of instruments should always be employed. Care should be taken that no instrument, however blunt, should come in contact with the cornea.

To acquire skill, you should dissect the muscles of the eye, in reference to this operation, as much as possible, and practice the fingers frequently in the use of the necessary instruments.

Before proceeding to an operation in any case, it is necessary that the diagnosis should be well established. The only affection you would often be liable to confound with ordinary strabismus is paralysis of the internal or external rectus, by which, of course, the cornea is permanently turned in the direction of the unaffected muscle by the tonic contraction of the latter. To the careless observer, paralysis of the external rectus might be mistaken for internal strabismus, since the eye (cornea) would, in such case, be turned towards the inner angle. *Vice versa* might (paralysis of the internal rectus) be confounded with external strabismus, since the cornea, in both cases, would be turned toward the external angle.

Spasmodic contraction of the internal or external rectus, which would, to a certain extent, simulate internal or external strabismus respectively, are very rare affections.

In reference to diagnosis, you should impress upon your minds the fact, that uncomplicated strabismus is a deviation of the visual axis (cornea) in a particular direction, to a degree, in each individual case, almost constant, with, however, almost perfect freedom of movement of the globe in all directions.

In the vast majority of cases, strabismus is an affection principally of the internal and external recti. You will rarely meet with superior and inferior strabismus.

In rotating the eyes from left to right, in uncomplicated strabismus, the corneæ both move to an equal extent, as you can observe by watching the distance over which the centre of the cornea, or a spot on the iris, moves from right to left along the edge of the lids.

The strabismus is sometimes alternately first in one eye then in the other. In either single or alternate strabismus, if one eye be fixed upon an object directly in front of the patient, the other eye will be "crossed;" if now the "straight" eye be covered with the hand, the "crossed" eye can be fixed upon the object. On inspecting the eye behind the hand, it will be found that this is now "crossed."

This form of strabismus has been termed of late years, "concomitant" strabismus, to designate it from fixed strabismus, (lucitas.)

When there is free movement of the eye, or when, on covering an eye already fixed upon an object, the "crossed" eye becomes straight, and the covered eye becomes crossed, there is almost always a favorable prognosis in regard to the result of the operation.

You should, however, bear in mind what many seem to forget, that, when the strabismus is very extensive, an operation often but partially restores the eye straight; or, if the eye becomes straight, that the other eye becomes turned. In such cases, it generally becomes necessary to operate at once on the other eye.

Although this operation is usually most satisfactory in respect to the restoration of the parallelism of the visual axes, and often improves vision, you must not expect that sight will always be regained in the affected eye—nor even that, without exception, the visual axis will be absolutely restored to its exact normal position.

The operation is, perhaps, attended with most favorable results, when the patient is between 5 and 15 years of age. I would advise you usually to perform the operation with the patient under the influence of an anæsthetic. You have seen, in two instances, how little dependance could be placed in the courage of patients, since, in these cases, the operation was interrupted by fainting.

The nature and causes of strabismus are still, in many respects, not well understood. Although long monographs have been written on the subject, the precise relation of cause and effect requires much more study. Donders, of Utrecht, after careful examination of many cases of convergent strabismus, has shown that, in a very large portion of instances, it is produced by hypermetropia, or that condition, as we have seen, in which the rays of light from very distant objects (parallel rays) are brought to a focus behind the retina, unless a certain portion of the power of accommodation is called into action.

You remember that the normal eye receives perfect images on the retina from very distant objects, when the accommodative apparatus is in perfect relaxation. Distinct vision of near objects with both eyes takes place, not only by calling in action the power of accommodation, but also by a contraction of the internal recti. There is a relation within certain limits between the amount of the accommodative power necessary in vision and the degree of convergence in the visual axes, produced by the contraction of the internal recti.

In the normal eye, on looking at very distant objects, the internal recti and the apparatus of accommodation are in perfect relaxation. As the eyes are directed to nearer objects, the divergency of the optic axes, as also the amount of contraction of the adjusting (ciliary) muscle, both increase in their relative proportion. The greater the demand upon the power of accommodation, (as in looking at nearer and nearer objects,) the greater is the tendency to converge the visual axes. Now in hypermetropic individuals, while looking at distant objects, the eyes (visual axes) remain parallel, while the power of accommodation is called into action. A portion of the accommodative power has thus been already necessarily expended before there is a demand upon the internal recti to produce convergency of the visual axes; it follows then, that, in looking at near objects, almost the whole power of accommodation is expended; and on looking at objects somewhat nearer, there is a necessity of the greatest possible effort on the part of the adjusting muscle. This great effort in the normal eye should be accompanied by a corresponding convergency of the visual axis.

But while the power of accommodation thus called into action in the hypermetropic eye would adapt the normal eye to a very near object, the required convergency is such as would be necessary in simply looking at quite distant objects. Such eyes are unable to endure this abnormal antagonism between the apparatus of accommodation and the recti muscles. Hence, while one eye is directed to the object, the undue tendency to contract in the other internal rectus, turns the eye (cornea) inward.

You will thus bear in mind that, in a very large proportion of cases of converging strabismus, the presence of hypermetropia is the predisposing cause.

On the other hand, myopia is the predisposing cause of diverging strabismus.

In the myopic eye, the antero-posterior diameter is so long, that the rays of light from distant objects, (the accommodating apparatus being totally relaxed,) unite at a point in front of the retina. The objects must be brought near the eye to form distinct images *on* the retina. Hence, as the object is near, although the apparatus of accommodation is in a state of relaxation, in which the visual axes should be parallel, there must be a convergency of these axes.

Now, as the eyes become fixed upon still nearer objects, there is a greater demand upon the internal recti for convergency, than is actually called for by the effort on the part of the accommodative apparatus. The force of the internal recti is thus partially expended before it is normally called for; it is all expended before the whole power of accommodation is called into action. As, with the increase in the effort of accommodation, more is demanded of the internal recti than they can perform, they become weary; the weaker muscle yields to its antagonist, the external rectus, and divergency is the result.

In addition to this cause, is one, perhaps, of more importance; the fact that the globe is elongated in myopia, increases the resistance to the action of the internal recti, and, consequently, the weaker muscle is sooner wearied, which tends to give a relatively greater power to one of the external recti.

I am well aware how imperfect these few remarks upon strabismus are. Our limited time has prevented a further discussion of the subject. I must, however, refer you to a few interesting and important points for your future study.

1st. The necessity of suitable convex lenses after operation for strabismus, where hypermetropia is present, not only to aid the vision of the patient but to prevent return of the strabismus.

2d. The fact that the line drawn from the centre of the cornea through the centre of the globe—or centre of motion, which is somewhat behind the mathematical centre, does not coincide with the line drawn from an image of an object on the macula lutea to the object, but forms an angle varying from about 5° to 9° and even 11°.

3d. Why all patients who are hypermetropic, are not affected with strabismus.

4th. What other conditions are favorable to the production of strabismus.

5th. The reason why, while paralysis of a muscle produces double vision, this is seldom present in uncomplicated strabismus; and why, sometimes after operating for strabismus, double vision appears.

6th. The indications for the operation of dividing the tendon of one of the recti, and bringing the end of the muscle forward, to be attached to the sclerotic nearer the cornea.

7th. The use of prismatic glasses.

Although much has been written on these subjects by different authors, I must refer you particularly to the works of Donders and Græfe.

